# Getting Started in Gene Expression Microarray Analysis

**DOI:** 10.1371/journal.pcbi.1000543

**Published:** 2009-10-30

**Authors:** Donna K. Slonim, Itai Yanai

**Affiliations:** 1 Department of Computer Science, Tufts University, Medford, Massachusetts, United States of America; 2 Department of Pathology, Tufts University School of Medicine, Boston, Massachusetts, United States of America; 3 Department of Biology, Technion–Israel Institute of Technology, Technion City, Haifa, Israel; Princeton University, United States of America

Gene expression microarrays provide a snapshot of all the transcriptional activity in a biological sample. Unlike most traditional molecular biology tools, which generally allow the study of a single gene or a small set of genes, microarrays facilitate the discovery of totally novel and unexpected functional roles of genes. The power of these tools has been applied to a range of applications, including discovering novel disease subtypes, developing new diagnostic tools, and identifying underlying mechanisms of disease or drug response. However, this technology necessarily produces a large amount of data, challenging us to interpret it by exploiting modern computational and statistical tools. In this brief review, we aim to indicate the major issues involved in microarray analysis and provide a useful starting point for new microarray users. Figure 1 outlines the steps in a typical expression microarray experiment and maps them to the different sections of this review.

**Figure 1:**
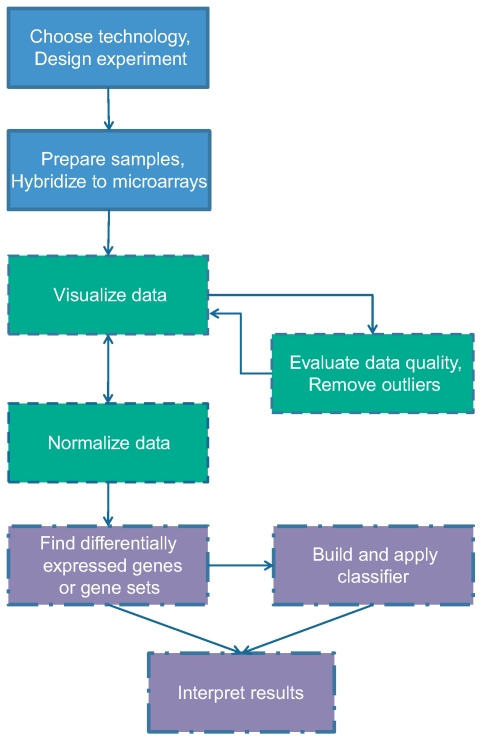
Overview of steps in a typical gene expression microarray experiment. Topics in blue boxes with solid borders are addressed in the Experimental Design section, those in green boxes with dashed borders are covered in the section on data preparation, and those in purple boxes with dash-dotted borders are discussed in the Data Analysis section of this review.

## Experimental Design

Careful experimental design is crucial for a successful microarray experiment [1],[2], yet this important step is often shortchanged. Design issues depend in part on the exact array technology used, and indeed, choosing an array technology is often the first design choice. The main distinction is whether essentially full-length transcripts are printed onto slides (cDNA microarrays) or the desired—typically shorter—oligonucleotides are synthesized in situ (oligonucleotide arrays). While the former may be less expensive because they can be manufactured in the lab or at institutional core facilities, the latter may outperform the former in terms of number of spots per array and the spots' homogeneity [3],[4].

Slightly different oligonucleotide array platforms are manufactured by companies such as Affymetrix, Agilent, and NimbleGen (see Text S1 and Table S1 for further discussion).

A major design question is whether to measure the expression levels from each sample on a different microarray (using single-color, or single-channel, arrays), or instead to compare relative expression levels between a pair of samples on each microarray (two-color or two-channel arrays). There are tradeoffs between the two approaches. Single-color arrays allow for more flexibility in analysis, while two-color arrays can control for some technical issues by allowing a direct comparison in a single hybridization [5]. A recent comparison of single- and two-color methods on the same platforms found good overall agreement in the data produced by the two methods [6]. cDNA arrays typically involve two channels. Agilent and NimbleGen arrays can be run using either one or two channels. Affymetrix arrays are inherently single-channel, though some associated analysis tools facilitate pair-wise comparisons.

Design issues for two-color arrays are more complex [7]. Challenges include ensuring that all samples can be compared to the appropriate controls and avoiding any biases introduced by the different labeling. “Dye-swap” experiments, in which the same pairs of samples are compared twice with the labeling colors swapped, can permit the computational removal of such bias. Dye swapping imposes additional costs in both the number of arrays and the types of data analyses possible. However, clever design can somewhat reduce the required number of arrays [1].

As attractive as it might seem financially to run just one microarray for each “class” of samples (of the same phenotype, time-point, or tissue type) under consideration, replicates are essential for providing meaningful results [2]. Without replicates, no statistical analysis of the significance and reliability of the observed changes is possible; the typical result is an increased number of both false-positive and false-negative errors in detecting differentially expressed genes [8]. However, we distinguish between technological and biological replicates. Technological replication—the same biological material hybridized independent times—is generally no longer performed, as analyses have shown that the results will be relatively consistent overall [4], although they may include consistent sources of bias [2]. Instead, different patients or animals from the same class can serve as biological replicates. To improve the ability to detect outliers and their effects, we do not recommend pooling samples unless necessary to obtain sufficient amounts of material for hybridization, and even then, replicates measuring different pools with the same phenotypes must be performed [7].

During the experimental design stage, it is important to identify all the variables to be compared and to ensure that the proposed design allows their measurement. Be aware of other variables, such as patient age or date of sample collection, that might confound the distinction between the compared classes. One option is to randomize confounding variables related to experimental conditions under your control.

## Preparing Microarray Data for Analysis

The task of analyzing microarray data is often at least as much an art as a science, and it typically consumes considerably more time than the laboratory protocols required to generate the data. Part of the challenge is assessing the quality of the data and ensuring that all samples are comparable for further analysis.

Normalization of the raw data, which controls for technical variation between arrays within a study, is essential [7]. The challenge of normalization is to remove as much of the technical variation as possible while leaving the biological variation untouched. This is a big challenge, and here we only touch upon the main issues. First, visualization of the raw data is an essential part of assessing data quality, choosing a normalization method, and estimating the effectiveness of the normalization. Many methods for visualization, quality assessment, and data normalization have been developed (see [9] for a review, Text S1, and Figure S1). Related issues of background adjustment and data “summarization” (reducing multiple probes representing a single transcript to a single measurement of expression) for Affymetrix arrays are well introduced in chapter 2 of [10].

Clustering is a way of finding and visualizing patterns in the data. Many papers and indeed books have been written on this topic (see e.g., [11]–[13] and Text S1). Different methods highlight different patterns, so trying more than one method can be worthwhile. Note that while clustering finds predominant patterns in the data, those patterns may not correspond to the phenotypic distinction of interest in the experiment. To identify gene expression patterns related to this distinction, more directed methods are appropriate.

## Data Analysis

There are many commercial packages for microarray analyses, and we have by no means evaluated all of them. However, commercial tools can be expensive, and we find many that we have tried to have limited flexibility. Fortunately, in the past few years a number of Web-based tools and open-source software packages for microarray data analysis have become available (see below and Text S1), and we recommend taking advantage of them.

One common strategy is to create a custom data analysis pipeline using statistical analysis software packages such as Matlab or R. Both allow great flexibility, customized analysis, and access to many specialized packages designed for analyzing gene expression data. Not only is R freely available, but it also allows the use of BioConductor [14], a collection of R tools including many powerful current gene expression analysis methods written and tested by experts from the growing microarray community.

The fundamental goal of most microarray experiments is to identify biological processes or pathways that consistently display differential expression between groups of samples. While the exact approach depends in part on the design of the experiment, there are two broad approaches to detecting differential expression. The first examines each gene or transcript individually to find genes that, by themselves, have statistically significant differences in expression between samples with different phenotypes or characteristics. The set of genes thus identified is then examined for over-representation of specific functions or pathways [15]. A powerful alternative is to identify groups of functionally related genes ahead of time and to test whether these gene sets—as a group—show differential expression [16]–[18]. Both of these approaches can be effective, and sometimes the combination of the two is stronger than either alone [19].

One crucial issue for all microarray analysis methods is adjusting for multiple testing [20]. Each statistical test reports the probability of seeing the observed test score by chance under the null hypothesis that there is no difference in expression related to the phenotype being studied. Even if this reported “*p*-value” is low, say 0.001, one might expect to see 20 of these one-in-a-thousand events when performing 20,000 independent tests (a reasonable number of genes on a microarray). A range of methods to adjust for multiple testing are available (see [21] for an overview). The preferred approach for microarray analysis is to control the “false-discovery rate” (FDR): the probability that any particular significant finding is a false positive [22].

Once a list of differentially expressed genes has been assembled, some functional analysis is essential for interpreting the results. There are many tools available to identify pathways or biological functions that are over-represented in a given gene list. Again, adjustment for multiple testing may be desirable, although complex dependencies between pathways make finding an appropriate adjustment method controversial [23]. A good review of the earlier tools that discusses many of the statistical issues is [15].

An alternative to the individual-gene analysis workflow is to consider entire gene sets or pathways together when looking for differential expression. There are many approaches that do this (e.g., [16], [24]–[26]), but a fundamental and widely used version is the Gene Set Enrichment Analysis (GSEA) software from the Broad Institute [17]. Gene set analysis can be advantageous because it can detect subtle changes in gene expression that individual gene analyses can miss, and because it combines identification of differential expression and functional interpretation into a single step.

The disadvantage of this method is that appropriate gene sets need to be known ahead of time. When studying a biological process that is still poorly understood, an individual gene method may be more appropriate, as it allows for the opportunity of implicating hitherto unexpected genes and gene sets. Given that gene set analysis is more sensitive and therefore potentially more powerful, a greater effort in defining the pathways needed to support this approach is warranted. Toward this end, GSEA's gene set database incorporates some computationally derived gene sets, including expression neighbors of known cancer genes [17] and network modules mined from a large collection of expression data [27]. Related work has used conserved coexpression [28] or differential coexpression [29] to discover new functional modules.

Much has also been written about sample classification using microarray data (see review [13]) but, with a few exceptions [30],[31], microarrays themselves have not been embraced as diagnostic tools. Rather, they have been used to identify smaller sets of predictive genes or pathways that might, when assessed by other technologies, aid in diagnosis or stratification of samples. A huge range of machine learning methods [11],[12] can be applied to the related classification problems. Most people intent on doing this write their own code (but see Text S1 for an alternative). We note that simpler classification tools often perform as well as, and generalize better than, more complex ones [32].

## Outlook

It has been our goal in this brief review to demonstrate that it is currently feasible for researchers with no previous experience to incorporate microarray analyses in their studies. The field is now reasonably mature, with available software and tools to make data analysis manageable by nonexperts. That said, newcomers to the field should be aware that the data analysis will require a dedicated commitment of time and effort that generally substantially exceeds that of data generation. We strongly recommend that researchers do the work to familiarize themselves with the relevant analytical literature before beginning, or even designing, the experiment.

It has been speculated that microarray technology will soon be superseded by next-generation sequencing, in which the transcripts are directly sequenced by low-cost, high-throughput sequencing technologies [33]. However, currently, next-generation whole-transcriptome sequencing is still quite expensive and in its relative infancy. Its cost scales proportionally with its ability to assess low-abundance transcripts, as sufficient depth of sequencing must be performed. Further, analytic tools specific to this data source have not yet been developed for mass consumption. Recent studies have shown that the two transcriptomics technologies are expected to give very similar results [34],[35], although for rare transcripts there is considerably less correlation between the methods [35]. Thus, until sequencing-based methods have become cost-effective and easily used, microarrays will remain a desirable alternative for many practitioners. We expect that, as RNA sequencing methods mature, many microarray analysis methods will come to be viewed as general analysis tools that can be applied or modified to fit any forthcoming transcriptomics technologies [36].

## Supporting Information

Figure S1 Three common normalization methods. The left plots show pairs of distributions of microarray intensities to be normalized (right plots). A) If the distributions are of the same overall shape, they can simply be scaled to the same mean. B) Quantile normalization imposes the same distribution on all samples. C) A known quantity of RNA is spiked-in to each sample (vertical line) and is then used as a scaling factor.
(1.57 MB EPS)

Text S1 In this section we further discuss some of the issues raised in the main text.
(0.23 MB RTF)

Table S1 Comparison of commercial microarray manufacturers.
(0.05 MB RTF)
