# Supplementary material for: Getting Started in Gene Expression Microarray Analysis
Source: PLoS Comput Biol. 2009 Oct 30;5(10):e1000543. doi: 10.1371/journal.pcbi.1000543 (PMC2762517; doi:10.1371/journal.pcbi.1000543)
Supplement: Text S1 — In this section we further discuss some of the issues raised in the main text. (0.23 MB RTF) [file pcbi.1000543.s002.rtf]

Supporting Information for “Getting Started in Gene Expression Microarray Analysis”, by Donna K. Slonim and Itai Yanai:

In this section we further discuss some of the issues raised in the main text.   To the extent that we make specific recommendations, we try to explain our reasoning.  Our priorities include data quality, statistical rigor, low or no cost, ease of use, and/or accessibility to biologists with limited programming experience.  

Choosing an oligonucleotide array

Among oligonucleotide platforms, we have the most experience with those produced by Affymetrix, Agilent, and Nimblegen.  Affymetrix microarrays are composed of spots of 25-bp probes. A target sequence is associated with a “probe-set,” typically 11-16 probes whose signal is integrated to produce a single intensity.  The sample is labeled by incorporation of biotin-labeled nucleotides, and a dedicated fluidics system washes the hybridized sample. Nimblegen and Agilent use different array synthesis methods that can create longer probes (up to ~ 60bp), and labeling is by cy3,5 fluores, which are also used to label cDNA arrays.  Table S1 compares these three oligonucleotide array technologies.

Most oligonucleotide array suppliers have catalogs of standard arrays for different species.  However, for designing custom microarrays, either for a new species not covered by standard microarrays or for a new application, Agilent's and Nimblegen's products are currently most affordable.

Single- or two-channel arrays?

An interesting paper [1] compares single- and two-channel methods on three platforms that allow for both options.  While it finds that the two choices perform similarly by many metrics, it suggests that two-channel comparisons may be slightly more sensitive and that single-channel designs may more accurately estimate fold-changes.  Single-channel microarray data is thought to be better geared toward estimating raw transcript abundance [2-4], perhaps in part because there is no competition between samples for the same probes.  As mentioned in the main text, using two-channel arrays also causes additional design challenges and can limit options for downstream analysis.  Thus, for the Nimblegen and Agilent technologies, where either choice is possible, we prefer using the single-channel option despite the apparent increase in the number of arrays needed (and hence cost).  However, note that any of these technologies can produce reliable and useful data [5], and that the care taken in the laboratory can dramatically affect the reliability of the data, often even more than the choice of platform [6].


Data preparation

There are three normalization methods that we use most frequently: mean-signal (or “scaling”), spike-in normalization, and quantile normalization (Figure S1).  Scaling is the simplest normalization method, in which each microarray's average expression level is set to the same value.  Though minimal, it avoids over-normalization, and may be effective if the samples to be compared can be expected to have similar average expression levels (e.g., they come from the same tissues and developmental stages, have similar mRNA quality, etc.).  The Affymetrix data analysis software allows for automatic array scaling [7]; samples can also be scaled manually using Excel, R, MatLab, or similar software.  

A more exacting method runs an internal control on each array by adding exogenous RNA of known quantities to the RNA sample.  These so-called “spike-in” methods can be valuable for comparing arrays where one cannot expect all samples to have the same average expression levels [8].  Agilent provides spike-in RNAs for this purpose[9]; Affymetrix incorporates suitable controls on their commercial arrays [8].  Spike-in controls are particularly helpful when comparing samples with inherently different transcriptomes; for example, a developmental time-course ranging from oocytes to tailbud stage larvae, which have vastly different amounts of mRNA.  In cases where spike-in controls are not necessary, or for pre-existing data where they were not part of the experiment, quantile normalization [10] is our current favorite among many popular normalization methods.  More strict than mean- or median-scaling, quantile normalization aligns the entire expression distributions on each array [10].  Thus, the most highly-expressed value is set to be the same across arrays, as is the next most highly-expressed, and so on.  RMA, another popular method, builds on this approach [11]; RMAexpress [12] offers an easy way to apply this method to Affymetrix arrays.
In addition to normalization of features across arrays, it has also been shown to be important to account for intra-array noise derived from such sources as dust on the slide, background surface variation, or scanning device biases.  This general error of measurement appears to vary greatly between datasets [13]. Several statistical models have been recently developed to mitigate these effects at the probe-level using model-based estimates [14-17] and are integrated in most of the  common normalization packages described here. The length to which researchers have gone to account for noise in microarray experiments highlights the importance of collecting many replicates to avoid being misled by noisy data. 

Clustering

There are at least as many clustering methods available as there are data sets, and nearly every expression analysis toolkit includes some clustering or data visualization tools.  We suggest trying a couple of different clustering methods to look at broad patterns in each data set and to ensure that such patterns don't correlate too obviously with any possible confounding variables (for example, we've seen samples cluster based on which lab technician performed the hybridization protocol!).  However, if the clusters simply don't make much sense, that's fine – just proceed with your analysis.  

Free clustering modules for R and Matlab are available online.  For users without the programming skills to work in these environments, the GenePattern software package from the Broad provides a full, freely available data analysis pipeline that includes a variety of clustering methods and data preparation tools [18].  Other clustering methods we like (though not all include software) include GeneSignature method [19], the two-way clustering method of Getz et al. [20], and the quality clustering method of Heyer et al.[21,22].   

Selecting differentially expressed genes

A wide range of methods to adjust for multiple testing are available [23].  Many rely on the assumption that the tests are independent, whereas we know that genes' expression patterns are heavily dependent on each other.  The preferred approach for microarray analysis is to control the “false-discovery rate” (FDR), the probability that any particular significant finding is a false-positive [24].  To better account for the dependencies within the data, we suggest multiple testing adjustment using “permutation-based” methods, which estimate the null distribution by permuting the actual data.  If that is not feasible, the Benjamini-Hochberg step-down method [25] offers a reasonable combination of statistical rigor and power for microarray analysis. 

The BioConductor [26] software package offers freely-available, customizable, and frequently-updated packages for identifying differential expression.  We recommend the multtest [27] and limma [28,29] packages in particular; both include FDR adjustment methods for multiple testing.  

Issues in choosing a differential expression method are nicely explained in [30].  Users of the multtest package can choose among several parametric methods (which make assumptions about the normality of the data), including the Welch t-test, paired t-test, or ANOVA [31].  All of these look for differences in the average expression level between groups.  Since assumptions about normality are often inappropriate [30],  the reported p-values are more appropriately used to as a guide to prioritizing the genes, not as accurate probabilities, even after adjusting for multiple testing.  Multtest also supports the non-parametric Wilcoxon method [27].  While more appropriate for non-normally distributed data, the power of this test is often significantly weaker, so important differential expression may be missed by this approach.  Sophisticated variance-estimation methods may be helpful when comparing small numbers of samples [32-34].   Non-programmers in particular may appreciate the SAM [34] Excel plug-in for finding differential expression, which adjusts for multiple testing, includes tools for handling time-course data, and is available at no cost to non-commercial users.  

The GenePattern analysis pipeline software [18]) is another freely available, stand-alone option that can help non-programmers identify individually differentially expressed genes using parametric tests and FDR adjustment.  GenePattern also performs sample classification using a number of standard machine learning methods, making it a good entry-point for biologists who want to classify new samples.   

Functional analysis of gene lists

For the R/Bioconductor user, the GOstats package [35] may be helpful in identifying over-represented GO terms among lists of differentially expressed genes.  Onto-Express [36,37] is a stand-alone tool that also finds significant GO term enrichment.  However,  GO terms are neither the only nor the best source of functional annotation available.  In fact, GO terms are assigned on a gene-by-gene or paper-by-paper basis, but not explicitly by pathway or function [38], so they are often ill-suited for defining functionally-related gene sets.  We have become increasingly fond of the web-based DAVID tools [39,40] for their ease of use and their large, customizable selection of available pathway databases.  

An alternative is gene set analysis.  GSEA is the gold standard here.  It comes in freely-available open-source Java, web-based, and R implementations, so it can be used by non-programmers (with a slight learning curve for formatting the data and understanding the program options), and can also be incorporated into R analysis pipelines or tweaked by programmers willing to dive into the code internals.   Permutation-based adjustment for multiple testing is performed.  Ranking the genes still relies on an individual test for differential expression, but several choices are provided in the software, or one can upload a ranked gene list.  Extensions to the basic GSEA methodology are becoming plentiful; several are reviewed in [41].

References


1. Patterson TA, Lobenhofer EK, Fulmer-Smentek SB, Collins PJ, Chu TM, et al. (2006) Performance comparison of one-color and two-color platforms within the MicroArray Quality Control (MAQC) project. Nat Biotechnol 24: 1140-1150.
2. Carter MG, Sharov AA, VanBuren V, Dudekula DB, Carmack CE, et al. (2005) Transcript copy number estimation using a mouse whole-genome oligonucleotide microarray. Genome Biol 6: R61.
3. Dudley AM, Aach J, Steffen MA, Church GM (2002) Measuring absolute expression with microarrays with a calibrated reference sample and an extended signal intensity range. Proc Natl Acad Sci U S A 99: 7554-7559.
4. Hubbell E, Liu WM, Mei R (2002) Robust estimators for expression analysis. Bioinformatics 18: 1585-1592.
5. Shi L, Reid LH, Jones WD, Shippy R, Warrington JA, et al. (2006) The MicroArray Quality Control (MAQC) project shows inter- and intraplatform reproducibility of gene expression measurements. Nat Biotechnol 24: 1151-1161.
6. Irizarry RA, Warren D, Spencer F, Kim IF, Biswal S, et al. (2005) Multiple-laboratory comparison of microarray platforms. Nat Methods 2: 345-350.
7. Affymetrix (2006) Affymetrix Expression Console Software Version 1.0 User Guide. Santa Clara, CA: Affymetrix, Inc.
8. Hill AA, Brown EL, Whitley MZ, Tucker-Kellogg G, Hunter CP, et al. (2001) Evaluation of normalization procedures for oligonucleotide array data based on spiked cRNA controls. Genome Biol 2: RESEARCH0055.
9. Zahurak M, Parmigiani G, Yu W, Scharpf RB, Berman D, et al. (2007) Pre-processing Agilent microarray data. BMC Bioinformatics 8: 142.
10. Bolstad BM, Irizarry RA, Astrand M, Speed TP (2003) A comparison of normalization methods for high density oligonucleotide array data based on variance and bias. Bioinformatics 19: 185-193.
11. Irizarry RA, Hobbs B, Collin F, Beazer-Barclay YD, Antonellis KJ, et al. (2003) Exploration, normalization, and summaries of high density oligonucleotide array probe level data. Biostatistics 4: 249-264.
12. rmaexpress.bmbolstad.com.
13. Albers CJ, Jansen RC, Kok J, Kuipers OP, van Hijum SA (2006) SIMAGE: simulation of DNA-microarray gene expression data. BMC Bioinformatics 7: 205.
14. Li C, Wong WH (2001) Model-based analysis of oligonucleotide arrays: expression index computation and outlier detection. Proc Natl Acad Sci U S A 98: 31-36.
15. Karakach TK, Wentzell PD (2007) Methods for estimating and mitigating errors in spotted, dual-color DNA microarrays. Omics 11: 186-199.
16. Rocke DM, Durbin B (2001) A model for measurement error for gene expression arrays. J Comput Biol 8: 557-569.
17. Brody JP, Williams BA, Wold BJ, Quake SR (2002) Significance and statistical errors in the analysis of DNA microarray data. Proc Natl Acad Sci U S A 99: 12975-12978.
18. Reich M, Liefeld T, Gould J, Lerner J, Tamayo P, et al. (2006) GenePattern 2.0. Nat Genet 38: 500-501.
19. Ihmels J, Friedlander G, Bergmann S, Sarig O, Ziv Y, et al. (2002) Revealing modular organization in the yeast transcriptional network. Nat Genet 31: 370-377.
20. Getz G, Levine E, Domany E (2000) Coupled two-way clustering analysis of gene microarray data. Proc Natl Acad Sci U S A 97: 12079-12084.
21. Heyer LJ, Kruglyak S, Yooseph S (1999) Exploring expression data: identification and analysis of coexpressed genes. Genome Res 9: 1106-1115.
22. Baugh LR, Hill AA, Slonim DK, Brown EL, Hunter CP (2003) Composition and dynamics of the Caenorhabditis elegans early embryonic transcriptome. Development 130: 889-900.
23. Westfall PH, Young SS (1993) Resampling-based multiple testing : examples and methods for P-value adjustment. New York: Wiley. xvii, 340 p. p.
24. Reiner A, Yekutieli D, Benjamini Y (2003) Identifying differentially expressed genes using false discovery rate controlling procedures. Bioinformatics 19: 368-375.
25. Benjamini Y, Hochberg Y (1995) Controlling the False Discovery Rate - a Practical and Powerful Approach to Multiple Testing. Journal of the Royal Statistical Society Series B-Methodological 57: 289-300.
26. Gentleman RC, Carey VJ, Bates DM, Bolstad B, Dettling M, et al. (2004) Bioconductor: open software development for computational biology and bioinformatics. Genome Biol 5: R80.
27. Pollard KS, Dudoit S, van der Laan MJ (2004) Multiple testing procedures and applications to genomics. Berkeley, CA: University of California.
28. Smyth G (2005) Limma:  linear models for microarray data. In: Gentleman RC, Carey VJ, Dudoit S, Irizarry R, Huber W, editors. Bioinformatics and Computational Biology Solutions using R and Bioconductor. New York: Springer. pp. 397-420.
29. Smyth GK (2004) Linear models and empirical bayes methods for assessing differential expression in microarray experiments. Stat Appl Genet Mol Biol 3: Article3.
30. Ewens WJ, Grant GR (2005) Statis[t]ical methods in bioinformatics : an introduction. New York, N.Y.: Springer. xx, 597 p. p.
31. Zar JH (1999) Biostatistical analysis. Upper Saddle River, N.J.: Prentice Hall. 1 v. (various pagings) p.
32. Baldi P, Long AD (2001) A Bayesian framework for the analysis of microarray expression data: regularized t -test and statistical inferences of gene changes. Bioinformatics 17: 509-519.
33. Huber W, von Heydebreck A, Sultmann H, Poustka A, Vingron M (2002) Variance stabilization applied to microarray data calibration and to the quantification of differential expression. Bioinformatics 18 Suppl 1: S96-104.
34. Tusher VG, Tibshirani R, Chu G (2001) Significance analysis of microarrays applied to the ionizing radiation response. Proc Natl Acad Sci U S A 98: 5116-5121.
35. Falcon S, Gentleman R (2007) Using GOstats to test gene lists for GO term association. Bioinformatics 23: 257-258.
36. Draghici S, Khatri P, Martins RP, Ostermeier GC, Krawetz SA (2003) Global functional profiling of gene expression. Genomics 81: 98-104.
37. Khatri P, Draghici S, Ostermeier GC, Krawetz SA (2002) Profiling gene expression using onto-express. Genomics 79: 266-270.
38. Ontology G (2007) GO Annotation Standard Operating Procedures.
39. Dennis G, Jr., Sherman BT, Hosack DA, Yang J, Gao W, et al. (2003) DAVID: Database for Annotation, Visualization, and Integrated Discovery. Genome Biol 4: P3.
40. Hosack DA, Dennis G, Jr., Sherman BT, Lane HC, Lempicki RA (2003) Identifying biological themes within lists of genes with EASE. Genome Biol 4: R70.
41. Nam D, Kim SY (2008) Gene-set approach for expression pattern analysis. Brief Bioinform 9: 189-197.
