# Supplementary material for: Getting Started in Gene Expression Microarray Analysis
Source: PLoS Comput Biol. 2009 Oct 30;5(10):e1000543. doi: 10.1371/journal.pcbi.1000543 (PMC2762517; doi:10.1371/journal.pcbi.1000543)
Supplement: Table S1 — Comparison of commercial microarray manufacturers. (0.05 MB RTF) [file pcbi.1000543.s003.rtf]

	Affymetrix	Roche Nimblegen	Agilent 	
Technology	Photolithography with masks	Maskless photolithography	Inkjet technology 	
Number of Features	~6M	~1M	~1M	
Arrays per chip	1	1x385k, 4x72k, 12x135k	1x1M, 2x400k,4x180, 8x60k	
Length of Probes 
(nucleotides)	25	60	60	
Labeling of nucleotides	biotin	cy3,5	cy3,5	
Probes per gene	4 to 11 probes per probe-set	2 to 12* 	2 to 50*	
Custom Design of Microarrays	prohibitively expensive	yes	yes	

*assuming 20k targets	
